# DNA methylation as a marker for prenatal smoke exposure in adults

**DOI:** 10.1093/ije/dyy091

**Published:** 2018-05-31

**Authors:** Rebecca C Richmond, Matthew Suderman, Ryan Langdon, Caroline L Relton, George Davey Smith

**Affiliations:** MRC Integrative Epidemiology Unit, Population Health Sciences, Bristol Medical School, University of Bristol, Bristol, UK

**Keywords:** prenatal smoking, ALSPAC, DNA methylation, epigenetics, long-term, prediction, epigenome-wide association study, longitudinal

## Abstract

**Background:**

Prenatal smoke exposure is known to be robustly associated with DNA methylation among offspring in early life, but whether the association persists into adulthood is unclear. This study aimed to investigate the long-term effect of maternal smoke exposure on DNA methylation in 754 women (mean age 30 years); to replicate findings in the same women 18 years later and in a cohort of 230 men (mean age 53 years); and to assess the extent to which a methylation score could predict prenatal smoke exposure.

**Methods:**

We first carried out an epigenome-wide association analysis for prenatal smoke exposure and performed replication analyses for the top CpG sites in the other samples. We derived a DNA methylation score based on previously identified CpG sites and generated receiver operating characteristic (ROC) curves to assess the performance of these scores as predictors of prenatal smoke exposure.

**Results:**

We observed associations at 15 CpG sites in 11 gene regions: *MYO1G*, *FRMD4A*, *CYP1A1*, *CNTNAP2*, *ARL4C*, *AHRR*, *TIFAB*, *MDM4*, *AX748264*, *DRD1*, *FTO* (false discovery rate <5%). Most of these associations were specific to exposure during pregnancy, were present 18 years later and were replicated in a cohort of men. A DNA methylation score could predict prenatal smoke exposure (30 years previously) with an area under the curve of 0.72 (95% confidence interval 0.69, 0.76).

**Conclusions:**

The results of this study provide robust evidence that maternal smoking in pregnancy is associated with changes in DNA methylation that persist in the exposed offspring for many years after prenatal exposure.

**Key Messages**

- We investigated the long-term impact of maternal smoking in pregnancy on epigenetic changes in the offspring by assessing differences in DNA methylation levels in adulthood.
- We observed associations at 15 CpG sites in 11 gene regions; most of these associations were specific to exposure during pregnancy, were found to persist until at least 48 years and were present in both men and women.
- A prenatal smoking score, derived by combining methylation values, could adequately predict whether the mothers of the adults smoked during pregnancy.
- The results of this study provide robust evidence that maternal smoking in pregnancy is associated with changes in DNA methylation that persist in the exposed offspring for many years after their prenatal exposure.
- These findings could have useful applications in epidemiological studies, e.g. by using DNA methylation signatures as a biosocial archive for exposure when data on maternal smoking during pregnancy are absent.

## Introduction

Cigarette smoke exposure during pregnancy is an environmental stressor that has a profound effect on DNA methylation in the exposed offspring.^1^ Previous work has determined genome-wide changes in DNA methylation in response to smoke exposure *in utero*,^2^ with a recent epigenome-wide association study (EWAS) meta-analysis of methylation in newborn cord blood identifying over 6000 differentially methylated CpG sites (of which 568 CpG sites surpassed the strict Bonferroni threshold).^3^

It is of interest to investigate the persistence of methylation marks into later life, as this presents the opportunity to use methylation as an archive of historical exposure, particularly if methylation patterns can be robustly modelled over time.^4–6^ In addition, persistent changes in DNA methylation might mediate at least some of the associations between smoke exposure in pregnancy and later-life health outcomes.^7^

Several studies have identified prenatal smoke exposure associated changes in methylation in childhood and adolescence in global methylation,^8^ candidate gene^8–10^ and EWAS.^3^^,^^4^^,^^6^^,^^11–13^ However, few studies have investigated the persistence of methylation change into adulthood.^14–17^ Three such studies have been conducted in the New York City birth cohort, where prospectively assessed maternal smoking during pregnancy was found to be positively associated with global methylation in leukocytes of individuals at age 43 years assessed using a methyl acceptance assay,^14^ inversely associated with levels of *Sat2* methylation^15^ and most recently associated with methylation at 17 CpG sites on the Illumina Infinium HumanMethylation450 array,^17^ which remained even after adjustment for adult smoking status of the offspring. However, previous studies were limited by low power due to small sample sizes and a more comprehensive assessment of the long-term impact of prenatal tobacco smoke exposure on genome-wide methylation is warranted.

In this study, we aimed to assess the long-term impact of prenatal tobacco smoke on DNA methylation in the context of the Avon Longitudinal Study of Parents and Children (ALSPAC)^18^^,^^19^—a prospective birth cohort with data on reported maternal smoke exposure in pregnancy and genome-wide DNA methylation levels of offspring in adulthood. We first conducted an analysis to investigate associations between reported prenatal smoke exposure and DNA methylation in peripheral blood among women in ALSPAC (mean age 30 years). We next attempted to replicate prenatal smoking-associated DNA methylation differences in peripheral blood of the women 18 years later (mean age 48 years) and in men from the same study (the partners of these women) (mean age 52 years). Finally, we aimed to assess the extent to which a prenatal smoking score, based on methylation at CpG sites previously shown to be associated with prenatal smoke exposure, could predict whether the mothers of the ALSPAC women smoked during pregnancy.

## Methods

### Cohort and selection of participants

ALSPAC is a large, prospective cohort study based in the south-west of England. A total of 14 541 pregnant women resident in Avon, UK, with expected dates of delivery 1 April 1991 to 31 December 1992 were recruited and detailed information has been collected on these women and their offspring at regular intervals.^18^^,^^19^ The study website contains details of all the data that are available through a fully searchable data dictionary (http://www.bris.ac.uk/alspac/researchers/data-access/data-dictionary/). Written informed consent has been obtained for all ALSPAC participants. Ethical approval for the study was obtained from the ALSPAC Ethics and Law Committee and the Local Research Ethics Committees.

We examined offspring DNA methylation in relation to reported maternal smoking during pregnancy in ALSPAC using methylation data from the Illumina Infinium HumanMethylation450 (HM450) BeadChip assay (Illumina, San Diego, CA, USA). We used data from women enrolled ALSPAC (*n* = 754) in the main analysis and looked to replicate findings in the same women approximately 18 years later (*n* = 656) and in men enrolled in ALSPAC (*n* = 230).

### Prenatal exposure variables

After recruitment of pregnant women into the ALSPAC study, information was collected on both the women and their partners, including details of their mothers’ smoking behaviour. If the men and women reported that their mothers had smoked, they were asked whether their mothers had smoked when they were pregnant with them and were given the responses yes/no/don’t know from which to select. These data were analysed assuming that, for all those who said don’t know, their mothers did smoke during pregnancy, as has been done previously.^20^ We carried out further sensitivity analysis excluding those individuals who were unsure of their mothers’ smoking status during pregnancy and compared findings.

### DNA methylation assessment

We examined offspring DNA methylation in peripheral blood in ALSPAC men and women. As part of the Accessible Resource for Integrated Epigenomics Studies (ARIES) project,^21^ the HM450 BeadChip^22^ has been used to generate epigenetic data on 1018 mother–offspring pairs in the ALSPAC cohort. A web portal has been constructed to allow openly accessible browsing of aggregate ARIES DNA methylation data (ARIES-Explorer) (http://www.ariesepigenomics.org.uk/). Additional HM450 data have been generated on ALSPAC men (the partners of the women enrolled in ARIES) (*n* = 312). Details of sample handling and DNA methylation profiling are outlined in the Supplementary Material, available as Supplementary data at *IJE* online.

### Covariates

Maternal age at birth and head of household social class were included as covariates in these analyses, as they were found to be most strongly associated with smoking status during pregnancy in a previous study.^4^ In addition, 10 surrogate variables and cell count fractions were included as additional covariates to adjust for technical batch and cell-type mixture (see Supplementary Material, available as Supplementary data at *IJE* online, for further details), although we did not find strong evidence for associations between prenatal smoke exposure and derived cell types (Supplementary Table 1, available as Supplementary data at *IJE* online).

In order to evaluate the potential influence of own smoking, which might explain the persistence in methylation signatures associated with intrauterine exposure, information on the ALSPAC women’s own smoking status was obtained from a questionnaire administered at 18 weeks’ gestation. Women were asked whether they had smoked regularly pre-pregnancy, from which a dichotomous variable for any tobacco smoking before pregnancy was derived. To assess the impact of passive smoke exposure, women were also asked whether their partners smoked at the same time point and whether their fathers (as well as their mothers) had smoked previously.

For the replication analysis, information on the women’s smoking status was also gathered in a questionnaire administered approximately 18 years later. Furthermore, information on the partner’s smoking status was obtained from a questionnaire administered to the partners approximately 21 years after the pregnancy. At these later time points, the men and women were asked whether they currently smoked or whether they had smoked every day when a smoker in the past. From these data, a dichotomous variable for any previous tobacco smoking was derived.

### Statistical analysis

We first performed an epigenome-wide association analysis in our largest sample: the ALSPAC women with methylation measured in peripheral blood taken at the time of enrolment in the study (*n* = 754). CpG level methylation [untransformed β-values, which is the ratio of the methylated probe intensity and the overall intensity and ranges from 0 (no cytosine methylation) to 1 (complete cytosine methylation)] was regressed against prenatal smoke exposure (any maternal smoking during pregnancy) with adjustment for covariates (maternal age, parental social class, offspring age, top 10 SVs in the main model). We then assessed whether associations were robust to adjustment for own smoking status (including own smoking status as a further covariate and also running the EWAS stratified by own smoking status) and passive smoke exposure (reported partner smoking).

To evaluate the specificity of the intrauterine effect and potential bias in our findings due to the role of passive smoke exposure out of pregnancy or residual confounding, we conducted two negative control tests^23–25^: (i) performing a comparison of the associations between parental smoking (any paternal smoking and any maternal smoking) and methylation levels at the top CpG sites and (ii) comparing associations between maternal smoking outside of pregnancy and methylation levels with maternal smoking during pregnancy and methylation levels at the same sites.

We next performed replication analyses for the top CpG sites [false discovery rate (FDR) < 0.05] in the other samples, by conducting EWAS of maternal smoking in the ALSPAC women 18 years later and ALSPAC men, adjusted for the same covariates as in the main model for the ALSPAC women at enrolment, and performing a look-up of the CpGs identified in the main analysis. We also assessed the association between prenatal smoke exposure and methylation at the identified sites in cord blood in the ALSPAC cohort that has been investigated previously^3^^,^^4^ (Supplementary Table 2, available as Supplementary data at *IJE* online). We next performed Pearson’s correlation analysis in order to compare consistency in effect estimates between the main analysis and the replication analysis.

We also investigated the association between prenatal exposure to smoking and DNA methylation for the 568 CpG sites previously found to be robustly associated with prenatal smoke in a cord blood meta-analysis at Bonferroni significance (*n* = 6685),^3^ in each of the adult cohorts. We assessed the degree of inflation of association signal (lambda value) for these CpG sites compared with that seen genome-wide across the samples and performed a Wilcoxon rank sum test to assess enrichment.

Furthermore, we generated a DNA methylation score^26^ for prenatal smoking based on these independently identified 568 CpG sites and compared its ability to predict whether the mothers of the ALSPAC adults had smoked during pregnancy with a score based on 19 CpG sites that reached Bonferroni significance in an EWAS of prenatal smoking conducted in peripheral blood of older children (*n* = 3187)^3^ and a score for own smoking, consisting of 2623 CpG sites that reached Bonferroni significance in the largest EWAS of own smoking to date (*n*= 9389 current vs never smokers).^27^ Details of these studies and how the scores were generated are outlined in Supplementary Table 2, available as Supplementary data at *IJE* online, and the Supplementary Material, available as Supplementary data at *IJE* online.

We generated receiver operating characteristic (ROC) curves for the prenatal and own smoking methylation scores and calculated the area under the curve (AUC) in order to assess the performance of these predictors using the pROC package in R. Given the overlap of CpG sites associated with both prenatal smoking and own smoking (Supplementary Figure 1, available as Supplementary data at *IJE* online), we also assessed the performance of the prenatal smoking score adjusted for the own smoking score. We compared the AUC of pairs of ROC curves using the Delong test for difference computed by the roc.test function as part of the pROC package.

We also investigated the extent to which the prenatal smoking scores could predict maternal smoking in pregnancy independently of own smoking status. This was done by comparing methylation scores between four different groups of participants, determined based on their own smoking status and that of their mothers during pregnancy: non-smokers whose mothers never smoked in pregnancy, smokers whose mothers never smoked in pregnancy, non-smokers whose mothers smoked in pregnancy and smokers whose mothers smoked in pregnancy. Pairwise comparisons between the groups were performed using two-tailed *t*-tests and a *p*-value for difference in methylation scores obtained. In addition, a *p*-value for trend was obtained from the linear regression of the methylation score on smoking status, where the four groups were included in an ordered categorical variable.

Analysis was performed using Stata (version 14) and R (version 3.3.1).

## Results

The cohort-specific summary statistics for this analysis are presented in Table 1. The ALSPAC women in our main analysis had a mean age of 30 years, whereas their mean age at follow-up was 48 years and the ALSPAC men had a mean age of 53 years. Maternal ages at birth were similar between the ALSPAC men and women, as were parental social class and rates of prenatal smoke exposure. Rates of own smoking varied quite substantially, from 16.9% in the ALSPAC women at the first time point to 33.8% in the ALSPAC men. There was limited evidence for an association between prenatal smoke exposure and own smoking in the three study groups (Supplementary Table 3, available as Supplementary data at *IJE* online).

**Table 1.** Descriptive characteristics of participant groups in this study

	ALSPAC adult females (Time Point 1)	ALSPAC adult females (Time Point 2)	ALSPAC adult males
*N*	754	656	230
Maternal age at birth (years) (SD)	27.6 (5.7)	27.8 (5.7)	28.2 (5.7)
Social class (manual) (*N*, %)	347 (46.0)	301 (45.9)	92 (40.0)
Prenatal smoke exposure (yes) (*N*, %)	216 (28.7)	179 (27.3)	73 (31.7)
Age at follow-up (years) (SD)	30.3 (4.3)	48.1 (4.2)	53.4 (5.1)
Sample type(s)	Whole blood/white cells	White cells/PBLs	White cells/PBLs
Own smoking (*N*, %)^a^	127 (16.9)	144 (28.9)	75 (33.8)

a The sample size of individuals who reported own smoking was slightly smaller with *N* = 752, *N* = 498 and *N* = 222 for ALSPAC adult females (Time Point 1), ALSPAC adult females (Time Point 2) and ALSPAC adult males (Time Point 3), respectively.

We observed associations between 15 CpG sites and prenatal smoking exposure in women at age 30 at FDR < 5% and 9 CpG sites that surpassed Bonferroni correction (Table 2). These sites were located in 11 gene regions and all but 2 have been previously identified in EWAS for maternal smoking at birth^3^ and into later life^3^^,^^4^^,^^6^^,^^10–12^^,^^17^ and all agree on direction of effect (Supplementary Table 4, available as Supplementary data at *IJE* online).

**Table 2.** DNA methylation changes associated with prenatal smoke exposure in ALSPAC women (Time Point 1)

CpG site	Chromosome	Gene region	Position	Basic model (*N* = 754^a^)				Adjusted model^b^ (*N* = 752)			
				Effect size	Standard error	*P*-value	FDR	Effect size	Standard error	*P*-value	FDR
**cg22132788**	**7**	***MYO1G***	**45002486**	**0.062**	**0.008**	**1.70E-13**	**8.26E-08**	**0.058**	**0.008**	**9.77E-13**	**4.75E-07**
**cg12803068**	**7**	***MYO1G***	**45002919**	**0.111**	**0.015**	**9.82E-13**	**2.38E-07**	**0.097**	**0.014**	**1.71E-11**	**4.16E-06**
**cg11813497**	**10**	***FRMD4A***	**14372879**	**0.038**	**0.006**	**1.25E-10**	**2.02E-05**	**0.036**	**0.006**	**4.85E-10**	**7.85E-05**
**cg04180046**	**7**	***MYO1G***	**45002736**	**0.041**	**0.006**	**2.53E-10**	**3.07E-05**	**0.037**	**0.006**	**3.43E-09**	**3.33E-04**
**cg05549655**	**15**	***CYP1A1***	**75019143**	**0.007**	**0.001**	**1.21E-08**	**0.001**	**0.007**	**0.001**	**8.82E-10**	**1.07E-04**
**cg25949550**	**7**	***CNTNAP2***	**145814306**	**–0.005**	**0.001**	**1.48E-08**	**0.001**	**–0.004**	**0.001**	**2.41E-07**	**0.015**
**cg19089201**	**7**	***MYO1G***	**45002287**	**0.038**	**0.007**	**1.75E-08**	**0.001**	**0.034**	**0.007**	**1.88E-07**	**0.013**
**cg05204104**	**2**	***ARL4C***	**235403141**	**0.023**	**0.004**	**2.30E-08**	**0.001**	**0.023**	**0.004**	**4.12E-08**	**0.003**
**cg17924476**	**5**	***AHRR***	**323794**	**0.045**	**0.008**	**6.24E-08**	**0.003**	**0.040**	**0.008**	**5.33E-07**	**0.029**
cg11429111	5	*TIFAB*	134813329	0.022	0.004	2.62E-07	0.013	0.019	0.004	2.90E-06	0.108
cg08241939	1	*MDM4*	204700816	0.022	0.004	5.04E-07	0.022	0.019	0.004	3.40E-06	0.110
cg11641006	2	*AX748264*	235213874	0.040	0.008	5.62E-07	0.023	0.029	0.006	1.27E-06	0.051
cg15016771	2	*ARL4C*	235403218	0.008	0.002	1.01E-06	0.036	0.007	0.002	3.35E-06	0.110
cg22807681	5	*DRD1*	174622933	0.023	0.005	1.04E-06	0.036	0.023	0.005	1.02E-06	0.045
cg26681628	16	*FTO*	54210550	0.037	0.008	1.27E-06	0.041	0.029	0.006	8.94E-06	0.193

Effect size = difference in methylation level (beta) between adult offspring of smokers and non-smokers in pregnancy.

Entries in bold represent sites that surpassed the Bonferroni threshold.

a *N* = 216 smoked during pregnancy, *N* = 538 no smoking during pregnancy.

b Model includes adjustment for own smoking.

Associations at CpG sites located near *ARL4C* (cg05204104 and cg15016771), *MDM4* (cg08241939) and *DRD1* (cg22807681) appear to be novel, although the sites at *ARL4C* were present in the extended list of FDR significant sites in the previous cord blood EWAS.^3^ In contrast to findings in cord blood, where smoking during pregnancy has been associated approximately equally with hyper and hypomethylation,^3^ the majority (14 out of 15 CpGs) showed long-term hypermethylation in this analysis.

Whereas, in most EWAS for cord blood methylation, *AHRR* (cg05575921) is the CpG site most consistently associated with prenatal smoke exposure,^3^^,^^4^^,^^28^^,^^29^ this site did not survive adjustment for multiple tests in our persistence analysis (*p* = 0.0005, FDR > 5%). Rather, associations at four sites at *MYO1G* (cg22132788, cg12803068, cg04180046 and cg19089201) did survive multiple testing adjustment, with the *MYO1G* site cg22132788 having the strongest association (*P* = 1.7 × 10^–13^), which is consistent with findings in children and adolescents.^4^^,^^6^^,^^12^

Results were robust to the additional inclusion of derived cell counts as covariates, with 14 of the 15 CpG sites surpassing the FDR < 5% threshold and the other CpG site at *DRD1* (cg22807681) with *p* = 5.22 × 10^–6^ (Supplementary Table 5, available as Supplementary data at *IJE* online). In sensitivity analysis where those participants who were unsure of their mothers’ smoking status during pregnancy were removed (*n* = 651 remained in the analysis, *n* = 103 were excluded), 12 CpG sites were found to be associated with prenatal smoke exposure at FDR < 5% in this smaller dataset (Supplementary Table 6, available as Supplementary data at *IJE* online). Nine of these CpG sites were overlapping with those in the main analysis, with the remaining three CpG sites located in similar gene regions [*AHRR* (cg05575921), *TIFAB* (cg01952185) and *FRMD4A* (cg25464840)].

**Figure 1.**
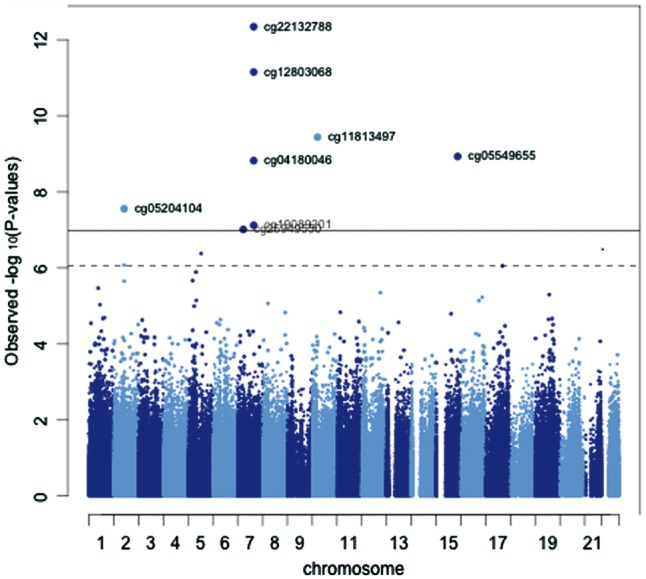
Manhattan plot for EWAS of prenatal smoke exposure in ALSPAC women (Time Point 1)*. *N = 752, adjusted for own smoking. Solid horizontal line represents Bonferroni threshold; dotted horizontal line represents FDR correction (*p* < 0.05) threshold.

One concern related to the identification of these signals is that they might reflect non-specific smoke exposure of the offspring over the life course rather than a ‘critical period’ effect of smoke exposure *in utero.*^30^ In particular, 11 out of the 15 CpGs have been identified in relation to current vs never smoking status at FDR < 0.05 in a recent EWAS of own smoking^27^ (Supplementary Table 4, available as Supplementary data at *IJE* online). To account for this, we first examined whether past cigarette smoking by the adult themselves influenced these associations by including own smoking as a covariate in the model. Adjustment for own smoking attenuated associations at five CpG sites [*TIFAB* (cg11429111), *MDM4* (cg08241939), *AX748264* (cg11641006), *ARL4C* (cg15016771) and *FTO* (cg26681628)] that no longer reached the FDR cut-off for significance (Table 2 and Figure 1), although, on the whole, the magnitude of effect was only slightly reduced with this adjustment. Furthermore, whereas methylation of CpG sites at *MYO1G*, *CNTNAP2* and *AHRR* have been consistently identified in relation to own smoking, no such associations have been found with CpG sites at *FDRM4A*, *CYP1A1*, *MDM4* or *DRD1* in the largest EWAS of own smoking to date.^31^ Findings were similar when analyses were stratified by own smoking status, i.e. with consistent effect estimates even among those women who had not previously smoked regularly themselves (Supplementary Table 7, available as Supplementary data at *IJE* online), although there was some evidence for a difference in effect sizes between smokers and non-smokers at *ARL4C* (cg05204104), where the effect was larger among smokers than among non-smokers (*p* for interaction = 0.001). Furthermore, results were consistent when reported partner smoking was included as a covariate as an indicator of passive smoke exposure (Supplementary Table 8, available as Supplementary data at *IJE* online).

For the negative control tests, the parental comparison showed consistently larger effect estimates for maternal smoking than for paternal smoking, although confidence intervals overlapped for some of the sites [*FRMD4A* (cg25464840), *CYP1A1* (cg05549655), *ARL4C* (cg05204104), *AHRR* (cg17924476) and *MYO1G* (cg04180046)] (Supplementary Figure 2, available as Supplementary data at *IJE* online). For the comparison of associations between the offspring of women who smoked during pregnancy and the offspring of women who smoked outside of pregnancy, effect estimates were consistently larger for those reporting maternal smoking during pregnancy, this time without overlapping confidence intervals (Supplementary Figure 3, available as Supplementary data at *IJE* online). Whereas, for *MYO1G* (cg12803066) and *MYO1G* (cg22132766), there was some evidence of association between maternal smoking outside of pregnancy with methylation levels, suggestive of either residual confounding in the intrauterine associations or a postnatal smoking effect, these findings may also be explained by misreporting of maternal smoking during pregnancy that was based on retrospective reports by the offspring in adulthood. This latter explanation is more likely given that the paternal smoking estimates at these sites were consistent with the null (Supplementary Figure 2, available as Supplementary data at *IJE* online).

**Figure 2.**
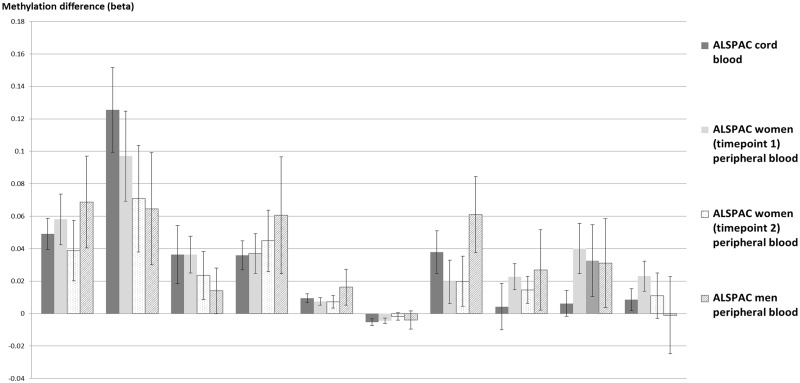
Replication of CpG sites observed below FDR (*p* < 0.05) threshold in ALSPAC women at a later time point (Time Point 2) and in ALSPAC men,* and comparison with effect of prenatal smoking on cord blood methylation in ALSPAC children. *Adjusted for own smoking in adult samples. *N* = 860 ALSPAC cord blood (reference), *N* = 752 ALSPAC women (Time Point 1), *N* = 498 ALSPAC women (Time Point 2), *N* = 222 ALSPAC men.

Effects at the top CpGs surpassing FDR correction in the ALSPAC women were found to be consistent in direction although slightly attenuated in the follow-up analysis approximately 18 years later (Pearson’s correlation coefficient, *r* = 0.92) (Figure 2 and Supplementary Figure 4, available as Supplementary data at *IJE* online). Furthermore, there was remarkable consistency in the direction and magnitude of effects at these CpGs in blood samples of the ALSPAC men (*r* = 0.77), with the exception of *DRD1*, where effects were not as consistently replicated. We also compared for reference the effect of any maternal smoking on cord blood methylation in the ALSPAC birth cohort at these CpGs and, again, both magnitude and direction of effect were similar (*r* = 0.92) with the exception of sites at *ARL4C* (cg05204104), *AHRR* (cg17924476) and *DRD4* (cg22807681). At these sites, the difference in methylation was greater in the ALSPAC women than in the newborns (Figure 2 and Supplementary Figure 4, available as Supplementary data at *IJE* online).

In addition, we found that, among women with a mean age of 30 years (*N* = 754), there was a strong signal of association above that expected by chance at CpG sites previously associated with prenatal smoke exposure in newborns [lambda = 2.62 vs 1.14 for all CpG sites on the Illumina Infinium HumanMethylation450 (HM450) BeadChip; Wilcoxon rank sum test *p*-value < 2.2 × 10^–16^] (Supplementary Figure 5, available as Supplementary data at *IJE* online). Similarly, inflation of signals for prenatal smoke exposure was seen in these women 18 years later (lambda = 1.54, *p*-value = 5.6 × 10^–15^) and in the ALSPAC men (lambda = 1.19, *p*-value = 2.2 × 10^–4^), compared with all CpG sites on the HM450 BeadChip (Supplementary Figure 5, available as Supplementary data at *IJE* online).

**Figure 3.**
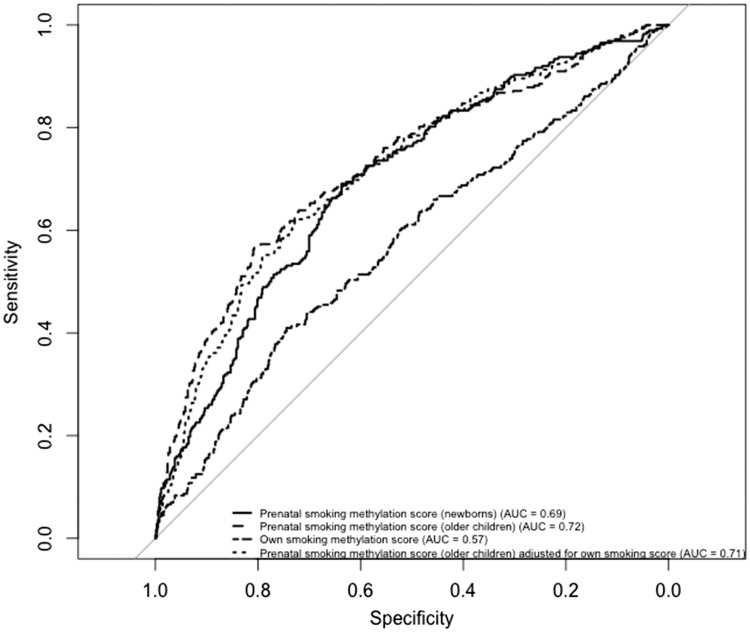
Receiver operating characteristic (ROC) curves of prenatal and own smoking methylation scores for discriminating maternal smoking in pregnancy. Total *N* = 922; NB: sample size in the main analysis is smaller due to inclusion of additional covariates into those models that were missing for some of the participants. Prenatal smoking methylation score (older children) = score derived from 19 CpG sites associated with maternal smoking in older children in an independent study.^3^ Prenatal smoking methylation score (newborns) = score derived from 568 CpG sites associated with maternal smoking in newborns in an independent study.^3^ Own smoking methylation score = score derived from 2623 CpG sites associated with smoking status in adults in an independent study.^31^ Scores were applied to methylation data from ALSPAC adult females at Time Point 1.

A prenatal smoking methylation score, derived by combining methylation values at 568 CpG sites associated with prenatal smoke exposure in cord blood of newborns in a previous study,^3^ could predict whether the mothers of the ALSPAC women smoked during pregnancy with an AUC 0.69 [95% confidence interval (CI) 0.67, 0.73]. This was comparable with a score derived from 19 CpG sites previously associated with prenatal smoking in peripheral blood of older children,^3^ which had an AUC 0.72 (95% CI 0.69, 0.76; *P* for difference = 0.97) (Figure 3).

To determine the extent to which methylation associations with prenatal smoking were different from own smoking associations, we constructed a similar score derived from 2623 CpG sites previously associated with own smoking in adulthood.^27^ Whereas being strongly predictive of own smoking status in the ALSPAC women (AUC 0.88, 95% CI 0.85, 0.91), this score was only weakly associated with prenatal smoking compared with the 19-CpG prenatal smoking score (AUC 0.57, 95% CI 0.53, 0.61; *P* for difference = 3.0 × 10^–11^) (Figure 3). In addition, the 19-CpG prenatal smoking methylation score was able to predict with the same accuracy prenatal smoke exposure when adjusted for the offspring smoking methylation score (AUC 0.71, 95% CI 0.68, 0.75; *P* for difference = 0.06) (Figure 3).

**Figure 4.**
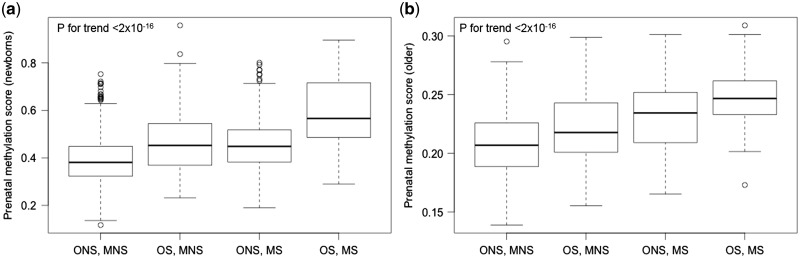
Box plots to assess differences in prenatal smoking methylation scores. Prenatal smoking methylation score (newborns) = score derived from 568 CpG sites associated with maternal smoking in newborns in an independent study.^3^ Prenatal smoking methylation score (older children) = score derived from 19 CpG sites associated with maternal smoking in older children in an independent study.^3^ ONS, MNS = offspring non-smoker, mother never smoked in pregnancy (*N* = 522); OS, MNS = offspring smoker, mother never smoked in pregnancy (*N* = 112); ONS, MS = offspring non-smoker, mother smoked in pregnancy (*N* = 222); OS, MS = offspring smoker, mother smoked in pregnancy (*N* = 66). NB: sample size in the main analysis is smaller due to inclusion of additional covariates into those models that were missing for some of the participants.

The prenatal smoking methylation scores were higher in individuals (both smokers and non-smokers) exposed to prenatal smoking compared with non-smokers who were not exposed prenatally (i.e. the ‘OS.MS’ and ‘ONS.MS’ group vs the ‘ONS.MNS’ baseline group; *p*-value for trend < 2.0 × 10^–16^) (Figure 4). The prenatal smoking methylation score derived from 568 CpG sites identified in newborns^3^ was not able to distinguish between non-smokers whose mothers smoked in pregnancy compared with smokers whose mothers did not smoke during pregnancy (difference in score between the ‘ONS.MS’ group and the ‘OS.MNS’ group = –0.07, *p* = 0.61) (Figure 4a). However, the score derived from 19 CpG sites which were shown to persist in relation to prenatal smoke exposure based on an EWAS in older children^3^ was higher in non-smokers whose mothers smoked in pregnancy compared with smokers whose mothers did not smoke in pregnancy (difference in score between the ‘ONS.MS’ group and the ‘OS.MNS’ group = 0.01, *p* = 0.001) (Figure 4b).

## Discussion

In a large longitudinal cohort with genome-wide methylation data, we identified 15 CpG sites that were differentially methylated in the peripheral blood of women over 30 years after exposure to prenatal smoking. Most of these signals remained in sensitivity analyses adjusted for own smoking and passive smoke exposure, and showed stronger associations in relation to maternal smoke exposure in pregnancy compared with smoke exposure outside of pregnancy (by both mothers and fathers) indicating specificity of the intrauterine effect. Furthermore, we observed a persistent methylation signal related to prenatal smoke exposure in peripheral blood 18 years later (i.e. at around the age of 48 years) and replicated in peripheral blood among men in the ALSPAC cohort.

Many of these CpG sites have been previously identified in relation to prenatal smoke exposure in the offspring at birth^3^ and the majority showed long-term hypermethylation among the offspring of smokers. Findings are also consistent with a recent report that highlighted persistence of DNA methylation levels related to prenatal smoke exposure into adulthood,^17^ which identified associations the same CpG sites located in *MYO1G* and *CYP1A1*, and other CpG sites in *FTO* and *AHRR*.

For all of the study samples, there was also a strong signal of association above that expected by chance at 568 CpG sites previously associated with prenatal smoke exposure in newborns from an independent study.^3^ In addition, we found that a prenatal smoking score, derived by combining methylation values at these CpG sites, could adequately predict whether the mothers of the adults in ALSPAC smoked during pregnancy with an AUC 0.69 (95% CI 0.67, 0.73). A recent study identified a much stronger predictive ability of a prenatal smoking methylation score with AUC of 0.90 in a test set of cord blood obtained from newborns in the MoBa cohort.^32^ The difference in predictive ability is therefore likely attributed to the 30-year difference in time since exposure and the generation of this methylation score using CpG sites identified in cord blood rather than adult peripheral blood. We also derived a methylation score based on CpGs that showed evidence of a persistent difference in methylation in peripheral blood of older offspring exposed to prenatal smoking^3^ who had a marginally higher AUC of 0.72 (95% CI 0.67, 0.73) and was also able to distinguish non-smokers whose mothers smoked in pregnancy from smokers whose mothers did not smoke during pregnancy.

Strengths of our study include the large sample size of women with reported maternal smoking in pregnancy for performing our initial EWAS analysis, the ability to adjust for own smoking status, the longitudinal assessment of differential methylation in a follow-up sample of these women and the replication analysis in men from the same study.

Although there was evidence of persistence for methylation differences even after adjusting for own smoking status in the adult offspring, there are limitations to performing this type of adjustment analysis. As parental smoking is strongly associated with their offspring’s smoking initiation,^33^ own smoking serves as a possible mediator on the path between prenatal smoking and offspring DNA methylation. This method of adjusting for a potential mediator in standard regression models to estimate the direct effect of an exposure may produce spurious conclusions.^34^^,^^35^ Whereas an alternative method of using life-course models previously provided more evidence for the hypothesis that maternal smoking in pregnancy is the ‘critical period’ for influencing persistent offspring methylation profiles,^4^ this method could not be applied here given the limited amount of information on maternal smoking reported by the ALSPAC men and women.

A further limitation relates to cell-type heterogeneity, given that the ALSPAC samples were obtained from a variety of sources [white cells, whole blood and peripheral blood lymphocytes (PBLs)]. To account for this, we incorporated surrogate variables into our models to account to adjust for technical batch and cell-type mixture in order to harmonize cellular variability of the samples^36^ and carried out sensitivity analysis that also adjusted for derived cell counts.^37^^,^^38^

In addition, information on prenatal smoke exposure in the ALSPAC men and women was recorded retrospectively by the adult offspring, rather than by prospective assessment, and so may be subject to more misreporting. Furthermore, in the ALSPAC men and women, rates of maternal smoking in pregnancy were reported to be high in comparison with contemporary populations. This draws to question the relevance of identified associations. However, we have shown that many of the signals identified in adults were also present in cord blood of offspring measured prospectively in a more contemporary cohort with lower rates of maternal smoking in pregnancy.^4^

Overall, the results of this study provide robust evidence that maternal smoking in pregnancy is associated with changes in DNA methylation that persist in the exposed offspring for many years after their prenatal exposure. Furthermore, these associations largely remain after adjusting for the previous smoking history of the adults themselves and are in accordance with earlier studies investigating prospectively assessed maternal smoking during pregnancy in relation to global DNA methylation levels.^14^^,^^15^

## Conclusion

These findings could have useful applications in epidemiological studies, e.g. by using DNA methylation signatures as a biosocial archive for historical exposure.^5^ Furthermore, persistent changes in DNA methylation might mediate at least some of the associations between smoke exposure in pregnancy and later-life health outcomes.^39^ However, distinguishing mediation from other association-driving mechanisms^40^ warrants further evaluation with the integration of analytical techniques such as two-step Mendelian randomization,^41^^,^^42^ transcriptomic analysis and the profiling of target tissues.^43^

## Funding

This work was supported by the Integrative Epidemiology Unit which receives funding from the UK Medical Research Council and the University of Bristol (MC_UU_12013_1 and MC_UU_12013_2). This work was also supported by CRUK (grant number C18281/A19169) and the ESRC (grant number ES/N000498/1). The UK Medical Research Council and the Wellcome Trust (Grant ref: 102215/2/13/2) and the University of Bristol provide core support for ALSPAC. The Accessible Resource for Integrated Epigenomics Studies (ARIES) was funded by the UK Biotechnology and Biological Sciences Research Council (BB/I025751/1 and BB/I025263/1). The methylation data generated on ALSPAC men (the partners of the women enrolled in ARIES) were funded by the Medical Research Council and the University of Bristol (MC_UU_12013_2). The funders had no role in study design, data collection and analysis, decision to publish or preparation of the manuscript.

## Supplementary Material

Supplementary Data
