# Supplementary material for: DNA methylation as a marker for prenatal smoke exposure in adults
Source: Int J Epidemiol. 2018 May 31;47(4):1120–30. doi: 10.1093/ije/dyy091 (PMC6124615; doi:10.1093/ije/dyy091)
Supplement: Supplementary Data [file dyy091_supplemental_material.docx]

# Supplemental Material

# DNA methylation as a marker for prenatal smoke exposure in adults

Rebecca C Richmond^1^*, Matthew Suderman^1^, Ryan Langdon^1^, Caroline L Relton^1±^, George Davey Smith^1±^

**Table of Contents**

**Supplementary Methods –** Sample handling and DNA methylation profiling; Covariates; DNA methylation scores

**Figure S1** - Venn diagram showing overlap of CpG sites from methylation scores

**Figure S2** - Comparison of associations between maternal and paternal smoking and offspring DNA methylation in adulthood

**Figure S3** - Comparison of associations between maternal smoking during pregnancy and maternal smoking not during pregnancy and offspring DNA methylation in adulthood

**Figure S4 -** Replication of CpG sites observed below FDR (p<0.05) threshold in ALSPAC women at a later time point and in ALSPAC men, and comparison with effect of prenatal smoking on cord blood methylation in ALSPAC children.

**Figure S5 -** QQ plots and lambda values for a) 568 CpG sites previously found to be robustly associated with prenatal smoke in a cord blood meta-analysis in ALSPAC b) all CpG sites in epigenome-wide association study

**Table S1 -** Estimated cell type proportions in peripheral blood of individuals whose mothers did and did not smoke during pregnancy

**Table S2 –** Epigenome-wide association studies contributing summary statistics for use in the current study

**Table S3 -** Cross-tabulation of own smoking and maternal smoking in study samples

**Table S4 -** Effect sizes from previous prenatal and own smoking epigenome-wide association studies

**Table S5** - DNA methylation changes associated with prenatal smoke exposure in ALSPAC women additionally adjusted for derived cell counts (Time Point 1, N=754)

**Table S6** - DNA methylation changes associated with prenatal smoke exposure in ALSPAC women, excluding those who were unsure of their mothers’ smoking status during pregnancy (Time Point 1)

**Table S7** - DNA methylation changes associated with prenatal smoke exposure in ALSPAC women stratified by own smoking status (Time Point 1)

**Table S8** - DNA methylation changes associated with prenatal smoke exposure in ALSPAC women adjusted for both own smoking and passive smoking (Time Point 1, N=709)

**References**

### **Supplementary Methods**

### **Sample handling and DNA methylation profiling**

The ARIES participants were selected based on the availability of DNA samples at two time points for the women (antenatal and at follow-up when the offspring were adolescents) and three time points for their offspring (neonatal, childhood [mean age 7.5 years], and adolescence [mean age 17.1 years]). Furthermore, additional HM450 data has been generated on ALSPAC men (the partners of the women enrolled in ARIES) (n=312).

Cord blood and peripheral blood samples were collected according to standard procedures, and the DNA methylation wet-laboratory and pre-processing analyses were performed as part of the ARIES project, as previously described (1). In brief, samples from all time points in ARIES were distributed across slides using a semi-random approach to minimize the possibility of confounding by batch effects. Samples failing quality control (average probe P value ≥0.01, those with sex or genotype mismatches) were excluded from further analysis and scheduled for repeat assay, and probes that contained <95% of signals detectable above background signal (detection P value <0.01) were excluded from analysis. Methylation data were pre-processed using R software, with background correction and subset quantile normalization performed using the pipeline described by Touleimat and Tost (2).

### **Covariates**

Information on the ALSPAC parents’ mothers’ age at delivery was recorded in a questionnaire administered at 18 weeks’ gestation. Information on the ALSPAC parents’ fathers’ social class was also recorded in the same questionnaire and was classified into non-manual or manual work. We also adjusted for current age at blood draw for methylation profiling among the participants. 10 surrogate variables (SVs) were generated using the “SVA” package in R and included in models to adjust for technical batch and cell-type mixture (3). To ensure cell counts were adequately captured, we further adjusted for derived fractions of CD8T-, CD4T-, NK- and B-cells, monocytes and granulocytes in a sensitivity analysis, using the *estimateCellCounts* function in the minfi Bioconductor package implemented in R (4, 5).

### **DNA methylation scores**

To derive the methylation scores, we used methylation data for the largest group of participants: the women in ALSPAC at the time of enrolment (n=754). For each individual, a weighted score was obtained by multiplying the methylation value at a given CpG by the effect size from the previous EWAS, and then summing these values:

b_1_cpg_1_ + b_2_cpg_2_ + … + b_n_cpg_n_

where “cpg” is the normalized methylation value in the ARIES women and “b” is the effect size from the aforementioned smoking EWAS (6, 7).

**Figure S1** - Venn diagram showing overlap of CpG sites from methylation scores

**
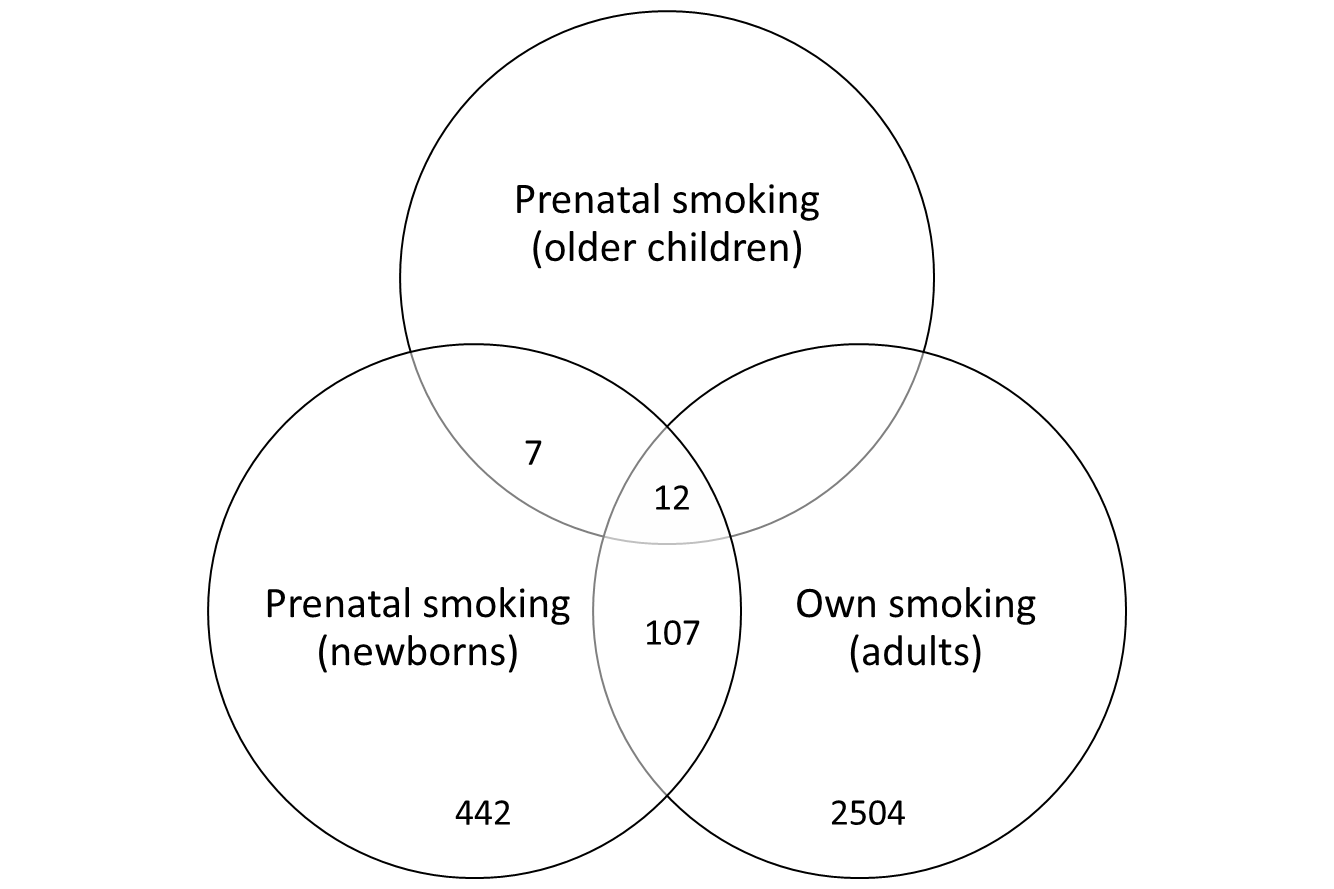
**

Prenatal smoking (older children) = methylation score derived from 19 CpG sites associated with maternal smoking in older children in an independent study (6). Prenatal smoking (newborns) = methylation score derived from 568 CpG sites associated with maternal smoking in newborns in an independent study (6). Own smoking (adults) = methylation score derived from 2623 CpG sites associated with smoking status in adults in an independent study (7).

**Figure S2** - Comparison of associations between maternal and paternal smoking and offspring DNA methylation in adulthood

**
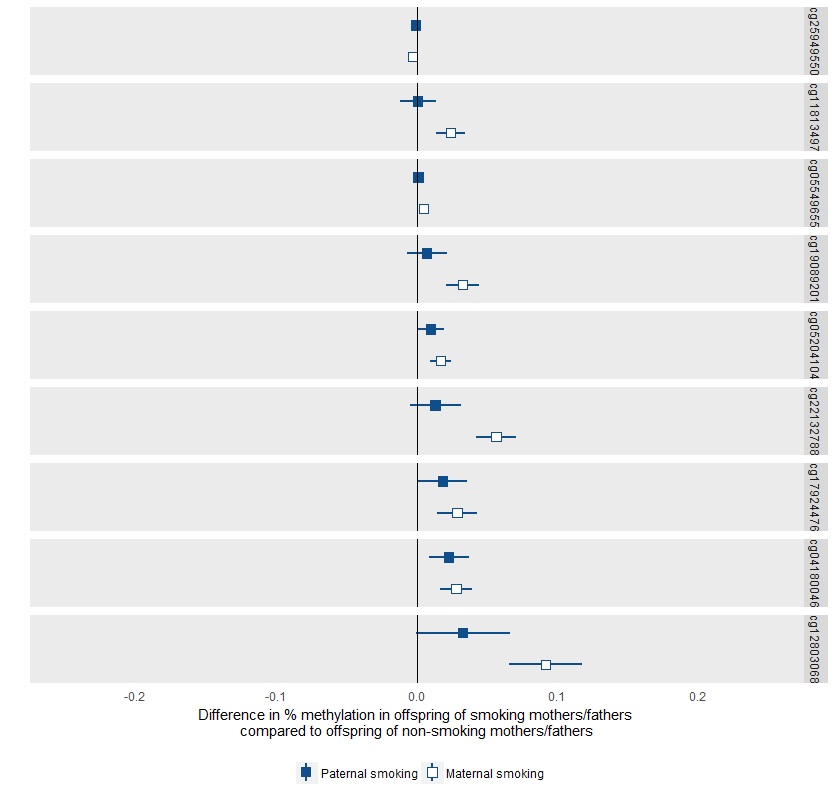
**

Ever smoking status of the mothers and fathers was recalled by the offspring (ALSPAC women) at recruitment into the study (Time Point 1). For paternal smoking: N = 192 never smoked, N = 542 ever smoked; for maternal smoking: N = 352 never smoked, N = 385 ever smoked. CpG sites associated with maternal smoking in pregnancy which surpassed Bonferroni correction were assessed.

**Figure S3** - Comparison of associations between maternal smoking during pregnancy and maternal smoking not during pregnancy and offspring DNA methylation in adulthood

**
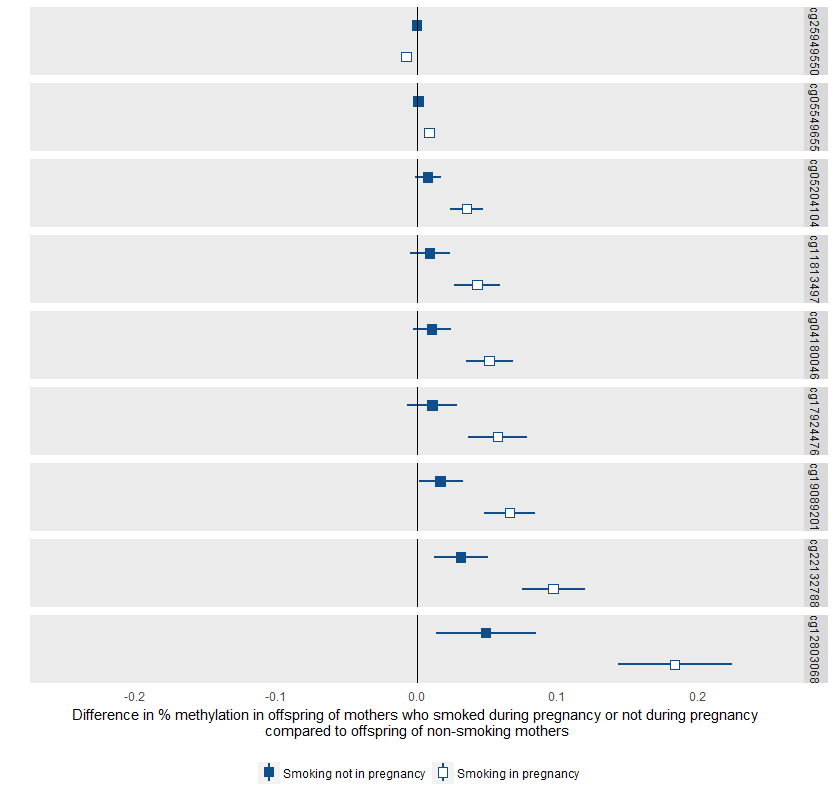
**

Smoking status of the mothers was recalled by the offspring (ALSPAC women) at recruitment into the study (Time Point 1) (previous smoking, and whether mothers had smoked when pregnant with them). For maternal smoking in pregnancy: N = 352 never smoked, N = 113 smoked during pregnancy; for maternal smoking not in pregnancy: N = 352 never smoked, N = 165 smoked but not during pregnancy. CpG sites associated with maternal smoking in pregnancy which surpassed Bonferroni correction were assessed.

**Figure S4 -** Replication of CpG sites observed below FDR (p<0.05) threshold in ALSPAC women at a later time point and in ALSPAC men*, and comparison with effect of prenatal smoking on cord blood methylation in ALSPAC children.


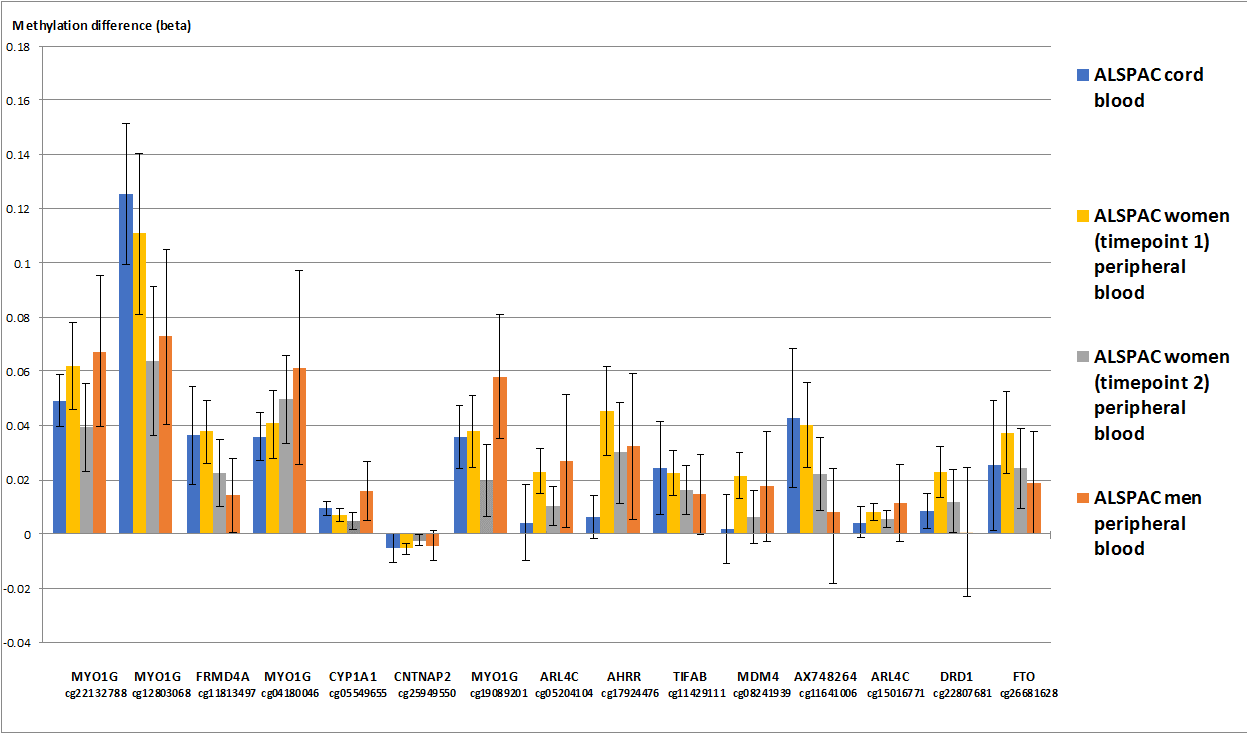


*not adjusted for current own smoking in adult samples. N= 860 ALSPAC cord blood (reference), N= 754 ALSPAC women (time point 1), N= 656 ALSPAC women (time point 2), N= 230 ALSPAC men

**Figure S5** QQ plots and lambda values for a) 568 CpG sites previously found to be robustly associated with prenatal smoke in a cord blood meta-analysis in ALSPAC* b) all CpG sites in epigenome-wide association study*


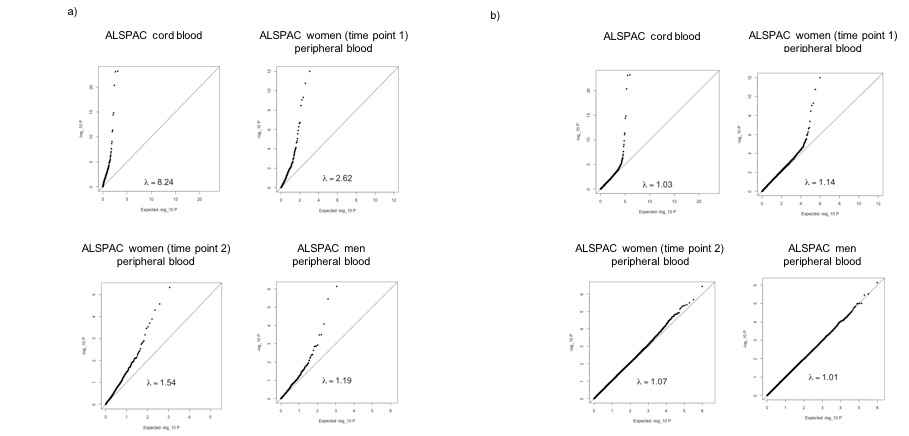


*adjusted for own smoking in adult samples. N= 860 ALSPAC cord blood (reference), N= 752 ALSPAC women (time point 1), N= 498 ALSPAC women (time point 2), N= 222 ALSPAC men

**Table S1 -** Estimated cell type proportions in peripheral blood of individuals whose mothers did and did not smoke during pregnancy

|  |  |  | |  |  |  |  |
| --- | --- | --- | --- | --- | --- | --- | --- |
|  | | | | | | | |
|  | Non-smoker (N=528) | | Smoked during pregnancy (N=212) | P value for difference |  |  |  |
| CD8+ T cells | 0.01 (0.02) | | 0.01 (0.02) | 0.73 |  |  |  |
| CD4+ T cells | 0.10 (0.05) | | 0.10 (0.04) | 0.48 |  |  |  |
| NK cells | 0.13 (0.04) | | 0.13 (0.04) | 0.70 |  |  |  |
| B cells | 0.07 (0.03) | | 0.07 (0.03) | 0.37 |  |  |  |
| Monocytes | 0.08 (0.03) | | 0.08 (0.03) | 0.09 |  |  |  |
| Granulocytes | 0.65 (0.08) | | 0.66 (0.07) | 0.63 |  |  |  |
|  |  | |  |  |  |  |  |
|  | | | | | | |  |

**Table S2 –** Epigenome-wide association studies contributing summary statistics for use in the current study

| Study | Cohort/consortium | PMID | Exposure | Outcome | Number of studies contributing results | Sample size | Tissue | Mean age of participant | Number of CpG sites identified which surpassed Bonferroni significance |
| --- | --- | --- | --- | --- | --- | --- | --- | --- | --- |
| Richmond et al, 2014 | Avon Longitudinal Study of Parents and Children (ALSPAC) | 25552657 | Prenatal exposure to maternal sustained smoking vs no smoking | DNA methylation (HM450*) at birth | 1 | 790 | Cord blood | 0 years | 15 |
| Joubert et al, 2016 | Pregnancy and Child Epigenetics Consortium (PACE) | 27040690 | Prenatal exposure to maternal sustained smoking vs no smoking | 1. DNA methylation (HM450*) at birth 2. DNA methylation (HM450*) in childhood | 1. 12 2. 5 | 1. 6, 685 2. 3, 187 | 1. Cord blood 2. Peripheral blood | 1. 0 years 2. 6.8 years | a) 568  b) 19 |
| Joehanes et al, 2016 | Cohorts for Health and Aging Research in Genomic Epidemiology Consortium (PACE) | 27651444 | Current vs never own smoking | DNA methylation (HM450*) in adulthood | 16 | 9389 | Peripheral blood | 62 years | 2,623 |

*HM450 = Illumina Infinium HumanMethylation 450 (HM450) BeadChip assay

**Table S3 -** Cross-tabulation of own smoking and maternal smoking in study samples

|  | | Maternal smoking | |
| --- | --- | --- | --- |
|  |  | No | Yes |
| Own smoking | No | 450 | 175 |
|  | Yes | 86 | 41 |

1. ARIES adult females at Time Point 1 (n= 752, Fisher’s exact p-value =0.33)
2. ARIES adult females at Time Point 2 (n= 498, Fisher’s exact p-value = 0.99)

|  | | Maternal smoking | |
| --- | --- | --- | --- |
|  |  | No | Yes |
| Own smoking | No | 262 | 92 |
|  | Yes | 107 | 37 |

1. Cross-tabulation of own smoking and maternal smoking in ARIES males (n= 222, Fisher’s exact p-value = 0.37)

|  | | Maternal smoking | |
| --- | --- | --- | --- |
|  |  | No | Yes |
| Own smoking | No | 103 | 44 |
|  | Yes | 48 | 27 |

**Table S4 -** Effect sizes from previous prenatal and own smoking epigenome-wide association studies*

| PMID | Table 1 Estimates | 27040690 | 27040690 | 27171005 | 25325234 | 25552657 | 28494218 | 27651444 |
| --- | --- | --- | --- | --- | --- | --- | --- | --- |
| Timepoint |  | Birth | Childhood | Childhood | Adolescence | Adolescence | Adulthood | Adulthood |
| Smoke exposure | Prenatal | Prenatal | Prenatal | Prenatal | Prenatal | Prenatal | Prenatal | Prenatal |
| cg22132788 | 0.06 | 0.02 | 0.04 | 0.05 | 0.06 | 0.09 | 0.07 | 0.05 |
| cg12803068 | 0.11 | 0.07 | 0.07 | 0.09 | 0.09 | NA | 0.11 | 0.06 |
| cg11813497 | 0.04 | 0.03 | 0.03 | 0.04 | NA | NA | NA | NA |
| cg04180046 | 0.04 | 0.05 | 0.04 | 0.05 | 0.07 | NA | 0.07 | 0.04 |
| cg05549655 | 0.01 | 0.02 | 0.01 | NA | 0.03 | 0.01 | 0.05 | NA |
| cg25949550 | -0.01 | -0.01 | -0.01 | -0.02 | –0.02 | -0.01 | NA | -0.02 |
| cg19089201 | 0.04 | 0.02 | 0.04 | 0.04 | 0.04 | NA | NA | 0.03 |
| cg05204104 | 0.02 | 0.01 | 0.02 | NA | NA | NA | NA | 0.02 |
| cg17924476 | 0.05 | 0.02 | 0.02 | NA | 0.03 | NA | NA | 0.03 |
| cg11429111 | 0.02 | 0.03 | 0.02 | NA | NA | NA | NA | 0.01 |
| cg08241939 | 0.02 | NA | NA | NA | NA | NA | NA | NA |
| cg11641006 | 0.04 | 0.02 | 0.01 | NA | NA | NA | NA | 0.01 |
| cg15016771 | 0.01 | 0.01 | 0.01 | NA | NA | NA | NA | 0.01 |
| cg22807681 | 0.02 | NA | NA | NA | NA | NA | NA | NA |
| cg26681628 | 0.03 | 0.02 | 0.02 | NA | NA | NA | NA | 0.01 |

*where effect estimates were available in published material

**Table S5** - DNA methylation changes associated with prenatal smoke exposure in ALSPAC women additionally adjusted for derived cell counts (Time Point 1, N=754)

| CpG site | Chromosome | Gene region | Position | Effect size | Standard error | P-value | FDR |
| --- | --- | --- | --- | --- | --- | --- | --- |
| cg22132788 | 7 | *MYO1G* | 45002486 | 0.061 | 0.008 | 1.35E-12 | 6.56E-07 |
| cg12803068 | 7 | *MYO1G* | 45002919 | 0.106 | 0.015 | 7.71E-12 | 1.87E-06 |
| cg11813497 | 10 | *FRMD4A* | 14372879 | 0.034 | 0.006 | 8.69E-09 | 7.47E-04 |
| cg04180046 | 7 | *MYO1G* | 45002736 | 0.036 | 0.007 | 7.15E-08 | 0.004 |
| cg05549655 | 15 | *CYP1A1* | 75019143 | 0.007 | 0.001 | 9.23E-09 | 7.47E-04 |
| cg25949550 | 7 | *CNTNAP2* | 145814306 | -0.006 | 0.001 | 1.52E-09 | 2.46E-04 |
| cg19089201 | 7 | *MYO1G* | 45002287 | 0.040 | 0.007 | 8.99E-09 | 7.47E-04 |
| cg05204104 | 2 | *ARL4C* | 235403141 | 0.024 | 0.004 | 1.22E-08 | 8.46E-04 |
| cg17924476 | 5 | *AHRR* | 323794 | 0.042 | 0.009 | 1.01E-06 | 0.034 |
| cg11429111 | 5 | *TIFAB* | 134813329 | 0.024 | 0.004 | 1.47E-07 | 0.008 |
| cg08241939 | 1 | *MDM4* | 204700816 | 0.022 | 0.004 | 1.33E-06 | 0.038 |
| cg11641006 | 2 | *AX748264* | 235213874 | 0.038 | 0.007 | 3.50E-07 | 0.015 |
| cg15016771 | 2 | *ARL4C* | 235403218 | 0.008 | 0.002 | 8.43E-07 | 0.033 |
| cg22807681 | 5 | *DRD1* | 174622933 | 0.022 | 0.005 | 5.22E-06 | 0.106 |
| cg26681628 | 16 | *FTO* | 54210550 | 0.037 | 0.008 | 1.06E-06 | 0.034 |

Effect size = difference in methylation level (beta) between adult offspring of smokers and non-smokers in pregnancy

*N= 216 smoked during pregnancy, N= 538 no smoking during pregnancy

**Table S6** - DNA methylation changes associated with prenatal smoke exposure in ALSPAC women, excluding those who were unsure of their mothers’ smoking status during pregnancy (Time Point 1)

| CpG site | Chromosome | Gene region | Position | Basic model (N=651) | | | |
| --- | --- | --- | --- | --- | --- | --- | --- |
|  |  |  |  | Effect size | Standard error | P-value | FDR |
| **cg22132788** | **7** | ***MYO1G*** | **45002486** | **0.089** | **0.010** | **5.96E-17** | **1.62E-11** |
| **cg12803068** | **7** | ***MYO1G*** | **45002919** | **0.158** | **0.018** | **6.68E-17** | **1.62E-11** |
| **cg19089201** | **7** | ***MYO1G*** | **45002287** | **0.059** | **0.008** | **7.36E-12** | **1.19E-06** |
| **cg25949550** | **7** | ***CNTNAP2*** | **145814306** | **-0.007** | **0.001** | **4.27E-10** | **5.19E-05** |
| **cg04180046** | **7** | ***MYO1G*** | **45002736** | **0.046** | **0.008** | **1.99E-09** | **1.93E-04** |
| **cg05204104** | **2** | ***ARL4C*** | **235403141** | **0.030** | **0.005** | **9.44E-09** | **7.63E-04** |
| cg05549655 | 15 | *CYP1A1* | 75019143 | 0.008 | 0.002 | 1.31E-07 | 0.009 |
| cg05575921 | 5 | *AHRR* | 373378 | -0.042 | 0.008 | 2.01E-07 | 0.012 |
| cg11429111 | 5 | *TIFAB* | 134813329 | 0.026 | 0.005 | 5.38E-07 | 0.029 |
| cg01952185 | 5 | *TIFAB* | 134813213 | 0.040 | 0.008 | 6.56E-07 | 0.032 |
| cg11813497 | 10 | *FRMD4A* | 14372879 | 0.037 | 0.007 | 9.14E-07 | 0.038 |
| cg25464840 | 10 | *FRMD4A* | 14372910 | 0.035 | 0.007 | 9.51E-07 | 0.038 |

Effect size = difference in methylation level (beta) between adult offspring of smokers and non-smokers in pregnancy

Entries in bold represent sites which surpassed the Bonferroni threshold

*N= 113 smoked during pregnancy, N= 538 no smoking during pregnancy

**Table S7** - DNA methylation changes associated with prenatal smoke exposure in ALSPAC women stratified by own smoking status (Time Point 1)

| CpG site | Chromosome | Gene region | Position | Smokers (N= 127*) | | | |  | | Non-smokers (N = 625†) | | | |
| --- | --- | --- | --- | --- | --- | --- | --- | --- | --- | --- | --- | --- | --- |
|  |  |  |  | Effect size | Standard error | P-value | Effect size | | Standard error | | P-value | Test for interaction |  |
| cg22132788 | 7 | *MYO1G* | 45002486 | 0.087 | 0.018 | 3.08E-06 | 0.052 | | 0.009 | | 2.96E-08 | 0.082 |  |
| cg12803068 | 7 | *MYO1G* | 45002919 | 0.142 | 0.034 | 4.62E-05 | 0.094 | | 0.017 | | 9.42E-08 | 0.207 |  |
| cg11813497 | 10 | *FRMD4A* | 14372879 | 0.038 | 0.015 | 0.011 | 0.035 | | 0.007 | | 1.87E-07 | 0.856 |  |
| cg04180046 | 7 | *MYO1G* | 45002736 | 0.053 | 0.019 | 0.007 | 0.033 | | 0.007 | | 2.21E-06 | 0.323 |  |
| cg05549655 | 15 | *CYP1A1* | 75019143 | 0.006 | 0.003 | 0.065 | 0.007 | | 0.001 | | 4.82E-08 | 0.752 |  |
| cg25949550 | 7 | *CNTNAP2* | 145814306 | -0.004 | 0.002 | 0.064 | -0.006 | | 0.001 | | 1.90E-07 | 0.371 |  |
| cg19089201 | 7 | *MYO1G* | 45002287 | 0.061 | 0.017 | 4.19E-04 | 0.034 | | 0.007 | | 7.77E-06 | 0.142 |  |
| cg05204104 | 2 | *ARL4C* | 235403141 | 0.056 | 0.011 | 3.52E-06 | 0.016 | | 0.005 | | 6.18E-04 | 0.001 |  |
| cg17924476 | 5 | *AHRR* | 323794 | 0.057 | 0.020 | 0.006 | 0.039 | | 0.009 | | 3.89E-05 | 0.412 |  |
| cg11429111 | 5 | *TIFAB* | 134813329 | 0.026 | 0.010 | 0.011 | 0.023 | | 0.005 | | 3.62E-06 | 0.788 |  |
| cg08241939 | 1 | *MDM4* | 204700816 | -0.001 | 0.011 | 0.960 | 0.026 | | 0.005 | | 3.49E-07 | 0.025 |  |
| cg11641006 | 2 | *AX748264* | 235213874 | 0.040 | 0.020 | 0.049 | 0.041 | | 0.009 | | 7.51E-06 | 0.964 |  |
| cg15016771 | 2 | *ARL4C* | 235403218 | 0.017 | 0.004 | 3.33E-05 | 0.006 | | 0.002 | | 8.85E-04 | 0.014 |  |
| cg22807681 | 5 | *DRD1* | 174622933 | 0.004 | 0.009 | 0.677 | 0.028 | | 0.005 | | 3.24E-07 | 0.020 |  |
| cg26681628 | 16 | *FTO* | 54210550 | 0.026 | 0.019 | 0.169 | 0.039 | | 0.009 | | 1.18E-05 | 0.536 |  |

Effect size = difference in methylation level (beta) between adult offspring of smokers and non-smokers in pregnancy

*N= 41 smoked during pregnancy, N= 86 no smoking during pregnancy; †N=175 smoked during pregnancy, N=450 no smoking during pregnancy

**Table S8** - DNA methylation changes associated with prenatal smoke exposure in ALSPAC women adjusted for both own smoking and passive smoking* (Time Point 1, N=709†)

| CpG site | Chromosome | Gene region | Position | Effect size | Standard error | P-value | FDR |
| --- | --- | --- | --- | --- | --- | --- | --- |
| cg22132788 | 7 | *MYO1G* | 45002486 | 0.055 | 0.009 | 2.17E-10 | 1.06E-04 |
| cg12803068 | 7 | *MYO1G* | 45002919 | 0.096 | 0.016 | 4.27E-09 | 0.001 |
| cg11813497 | 10 | *FRMD4A* | 14372879 | 0.036 | 0.006 | 7.65E-09 | 0.001 |
| cg04180046 | 7 | *MYO1G* | 45002736 | 0.036 | 0.007 | 1.69E-07 | 0.012 |
| cg05549655 | 15 | *CYP1A1* | 75019143 | 0.007 | 0.001 | 3.17E-08 | 0.003 |
| cg25949550 | 7 | *CNTNAP2* | 145814306 | -0.006 | 0.001 | 3.30E-08 | 0.003 |
| cg19089201 | 7 | *MYO1G* | 45002287 | 0.035 | 0.007 | 1.20E-06 | 0.050 |
| cg05204104 | 2 | *ARL4C* | 235403141 | 0.024 | 0.004 | 6.91E-08 | 0.006 |
| cg17924476 | 5 | *AHRR* | 323794 | 0.045 | 0.009 | 2.09E-07 | 0.013 |
| cg11429111 | 5 | *TIFAB* | 134813329 | 0.022 | 0.005 | 1.12E-06 | 0.050 |
| cg08241939 | 1 | *MDM4* | 204700816 | 0.021 | 0.005 | 7.73E-06 | 0.179 |
| cg11641006 | 2 | *AX748264* | 235213874 | 0.040 | 0.008 | 2.31E-06 | 0.080 |
| cg15016771 | 2 | *ARL4C* | 235403218 | 0.008 | 0.002 | 4.19E-06 | 0.118 |
| cg22807681 | 5 | *DRD1* | 174622933 | 0.025 | 0.005 | 5.71E-07 | 0.031 |
| cg26681628 | 16 | *FTO* | 54210550 | 0.038 | 0.008 | 4.22E-06 | 0.118 |

Effect size = difference in methylation level (beta) between adult offspring of smokers and non-smokers in pregnancy

*Model includes adjustment for own smoking and reported partner smoking

†N=201 smoked during pregnancy, N=508 no smoking during pregnancy

**References**

1. Relton CL, Gaunt T, McArdle W, Ho KR, Duggirala A, Shihab H, et al. Data Resource Profile: Accessible Resource for Integrated Epigenomic Studies (ARIES). Int J Epidemiol. 2015;44(4):1181-90.

2. Touleimat N, Tost J. Complete pipeline for Infinium((R)) Human Methylation 450K BeadChip data processing using subset quantile normalization for accurate DNA methylation estimation. Epigenomics-Uk. 2012;4(3):325-41.

3. McGregor K, Bernatsky S, Colmegna I, Hudson M, Pastinen T, Labbe A, et al. An evaluation of methods correcting for cell-type heterogeneity in DNA methylation studies. Genome Biol. 2016;17.

4. Houseman EA, Accomando WP, Koestler DC, Christensen BC, Marsit CJ, Nelson HH, et al. DNA methylation arrays as surrogate measures of cell mixture distribution. Bmc Bioinformatics. 2012;13.

5. Jaffe AE, Irizarry RA. Accounting for cellular heterogeneity is critical in epigenome-wide association studies. Genome Biol. 2014;15(2).

6. Joubert BR, Felix JF, Yousefi P, Bakulski KM, Just AC, Breton C, et al. DNA Methylation in Newborns and Maternal Smoking in Pregnancy: Genome-wide Consortium Meta-analysis. Am J Hum Genet. 2016;98(4):680-96.

7. Joehanes R, Just AC, Marioni RE, Pilling LC, Reynolds LM, Mandaviya PR, et al. Epigenetic Signatures of Cigarette Smoking. Circ Cardiovasc Genet. 2016;9(5):436-47.
